# The Effect of Simvastatin on the Dynamics of NF-κB-Regulated Neurodegenerative and Neuroprotective Processes in the Acute Phase of Ischemic Stroke

**DOI:** 10.1007/s12035-023-03371-2

**Published:** 2023-05-19

**Authors:** Grazyna Lietzau, Waldemar Sienkiewicz, Zbigniew Karwacki, Jerzy Dziewiątkowski, Jerzy Kaleczyc, Przemysław Kowiański

**Affiliations:** 1 Division of Anatomy and Neurobiology, Faculty of Medicine, Medical University of Gdańsk, Dębinki 1, 80-211 Gdańsk, Poland; 2 Department of Animal Anatomy, Faculty of Veterinary Medicine, University of Warmia and Mazury, Oczapowskiego 13, 10-719 Olsztyn, Poland; 3 Department of Neuroanaesthesiology, Faculty of Medicine, Medical University of Gdańsk, Dębinki 7, 80-211 Gdańsk, Poland; 4 Institute of Health Sciences, Pomeranian University in Słupsk, Bohaterów Westerplatte 64, 76-200 Słupsk, Poland

**Keywords:** Ischemic stroke, NF-κB, Neurodegeneration, Neuroprotection, Statins

## Abstract

Statins are lipid-lowering drugs that act by inhibiting 3-hydroxy-3-methylglutaryl coenzyme A reductase, a rate-limiting enzyme in cholesterol biosynthesis. Animal studies have shown neuroprotective effects of statins in cerebral stroke. However, the underlying mechanisms are not fully understood. The nuclear factor-kappa B (NF-κB) transcription factor is involved in the regulation of apoptosis in stroke. Different dimers of NF-κB regulate the gene expression of proteins involved in both neurodegeneration and neuroprotection. We aimed to determine whether simvastatin improves stroke outcome via inhibition of the RelA/p65-containing subunit and downregulation of stroke-induced pro-apoptotic genes or via activation of NF-κB dimers containing the c-Rel subunit and upregulation of anti-apoptotic genes during the acute stroke phase. Eighteen-month-old Wistar rats, subjected to permanent MCAO or sham surgery, were administered simvastatin (20 mg/kg b.w.) or saline for 5 days before the procedure. Stroke outcome was determined by measuring cerebral infarct and assessing motor functions. The expression of NF-κB subunits in various cell populations was investigated using immunofluorescence/confocal microscopy. RelA and c-Rel were detected by WB. The NF-κB-DNA binding activity was investigated using EMSA, and expression of *Noxa*, *Puma*, *Bcl-2*, and *Bcl-x* genes was analyzed by qRT-PCR. Results showed a 50% infarct size reduction and significant motor function improvement in the simvastatin-treated animals which correlated with a decrease in RelA and a transient increase in the c-Rel level in the nucleus, normalization of the NF-κB-DNA binding activity, and downregulation of the NF-κB-regulated genes. Our results provide new insights into the statin-mediated neuroprotective action against stroke based on NF-κB pathway inhibition.

**Supplementary Information:**

The online version contains supplementary material available at 10.1007/s12035-023-03371-2.

## Introduction

Statins are a class of drugs approved and commonly used for dyslipidemia treatment. By inhibiting 3-hydroxy-3-methylglutaryl coenzyme A (HMG-CoA) reductase, they limit the hepatic synthesis of cholesterol and its derivatives, which together with an increased uptake of low-density lipoprotein (LDL) in the liver leads to a reduction of total cholesterol and LDL in the blood. They were also reported to reduce triglycerides and increase high-density lipoprotein (HDL) levels [1, 2]. It is well-established that pharmacological regulation of lipid metabolism with statins in patients with dyslipidemia significantly reduces the risk of cerebral stroke morbidity [3–8], mortality [9–11], and its recurrence [8, 12–14]. However, the abovementioned clinical benefits of statin use in stroke result not only from their lipid-lowering properties but also from their cholesterol-independent (pleiotropic) effects. These include modulation of cell signaling pathways, vascular endothelial function improvement, enhancement of the stability of atherosclerotic plaques, reduction of oxidative stress, inflammation, and angiogenesis, as well as vasodilatation and inhibition of the thrombogenic response (reviewed in [15, 16]). Numerous animal stroke studies have demonstrated the neuroprotective effects of statins, as manifested by a reduction in cerebral infarct size, decreased edema and blood–brain barrier (BBB) damage, and improvement of functional outcomes (reviewed in [17]).

One of the pathways involved in the evolution of stroke infarction and affected by statin treatment is the nuclear factor kappa-light-chain-enhancer of activated B cells (NF-κB)-mediated pathway. NF-κB is a transcription factor sequestered in the cytoplasm where it is bound to inhibitory proteins known as the inhibitors of NF-κB (IκB). Stimuli activate an upstream kinase (IκB kinase) which results in the phosphorylation of IκB leading to its ubiquitination and proteasomal degradation. IκB degradation releases NF-κB, which translocates from cytoplasm to the nucleus and induces gene expression. This process is terminated through NF-κB-induced IκB synthesis followed by cytoplasmic resequestration [18, 19]. The role of NF-κB is not fully understood due to its diversity and the complex regulation of many central nervous system (CNS) processes, both under physiological and pathological conditions. Under physiological conditions, this protein complex has a low level of activity in neurons, glial cells, and cerebrovascular endothelial cells [20]. It controls DNA transcription, cytokine production, and cell survival [21]. More recent studies suggest that NF-κB is also involved in neurogenesis, neuritogenesis, synaptic plasticity, and learning and memory processes [22–27]. Under ischemic conditions, activated NF-κB was reported to promote both cell death [28, 29] and neuronal survival [30, 31]. Various actions of NF-κB in stroke and other brain pathologies may depend on the NF-κB complex composition [32]. NF-κB complexes are different combinations of Rel family protein dimers that include RelA/p65, p52, p50, RelB, and c-Rel [33, 34]. While complexes containing the RelA subunit, inducing transcription of a panel of pro-apoptotic genes, are mainly responsible for the stroke-triggered neurodegeneration [20, 35], activated c-Rel-containing NF-κB dimers participate in anti-apoptotic gene regulation and counteract ischemic damage by acting as an innate neuroprotective mechanism [36].

In addition, the opposite effects of NF-κB have been reported in different CNS cell populations. A growing body of evidence supports the anti-apoptotic role of NF-κB in neurons. However, prolonged activation of this transcription factor in reactive glial cells has been associated with detrimental outcomes, inflammation, and neuronal death (reviewed in [37]). The role of NF-ĸB in the latter is a result of both the induction of pro-apoptotic genes transcription and the inhibition of anti-apoptotic gene activity [38]. NF-ĸB regulates transcription of genes for Bcl-2 family proteins, which control cellular levels of Ca^2+^, K^+^, and Cl^–^ ions, redox, outflow of pro-apoptotic factors from mitochondria, and activation of key enzymes involved in apoptosis [39]. Among them are genes encoding anti-apoptotic proteins BCL-2 and BCL-XL and genes encoding pro-apoptotic BH3-only proteins NOXA and PUMA [35, 40–42]. Whether NF-ĸB promotes or inhibits apoptosis is thought to depend on the type and duration of the stimulus, cell population, and region of the brain [20, 43].

A few reports have suggested an inhibitory effect of statins on the NF-κB pathway in ischemic stroke [44, 45]. However, in these studies, the efficacy of statins was only assessed at one time point, and no data are available on the effects of statins on the pro- and anti-apoptotic processes regulated by this transcription complex and on how the neuroprotection–neurodegeneration dynamics is shaped in the acute phase of stroke. The goal of this study was to determine the effect of short-term pretreatment with simvastatin (ST) on NF-κB activation and expression of the pro- and anti-apoptotic genes regulated by different subunits of this transcription complex in the infarct (striatum) and peri-infarct (frontoparietal cortex) areas during the first 24 h of an ischemic stroke without reperfusion.

## Materials and Methods

### Animals and Experimental Design

**Fig. 1:**
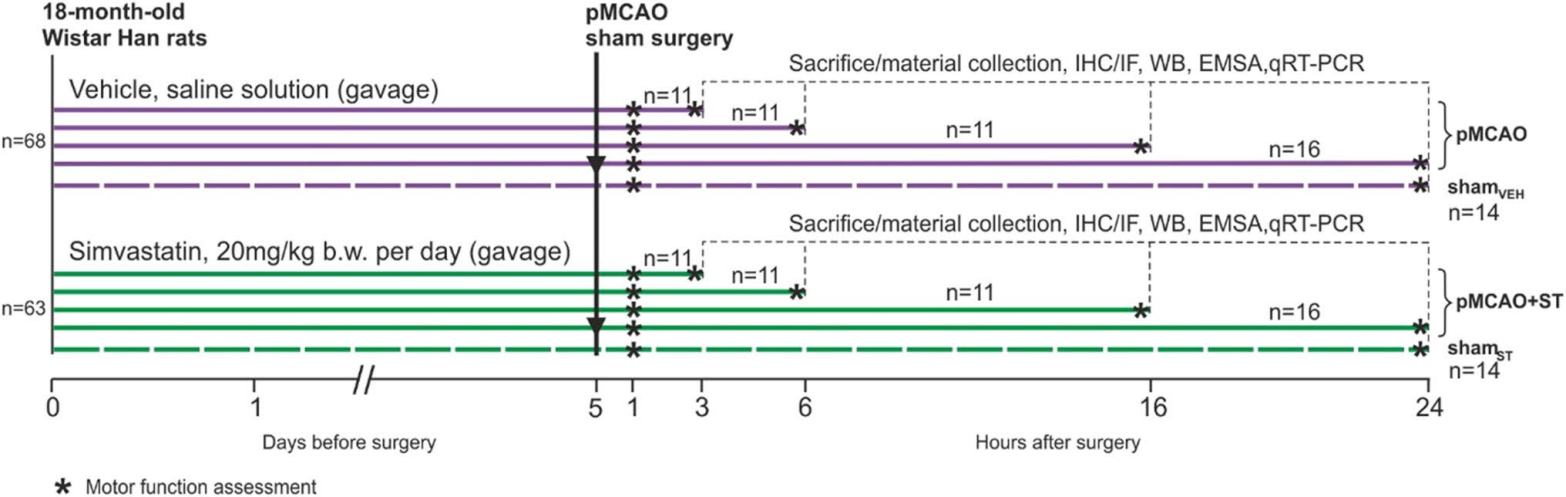
Experimental timeline and drug–treatment paradigm

Eighteen-month-old male Wistar Han rats (Tri-City University Animal House—Research Service Centre, Poland) were housed in controlled conditions (22–21 °C, 45–65% relative air humidity, air was exchanged 15–20 times per hour, 12-h light/dark cycle) with free access to food and water. Initially, simple randomization method was used to allocate the rats to two experimental groups: 1) vehicle group (*n* = 63) receiving saline solution (gavage feeding) and 2) simvastatin (ST, Polfarmex S.A., Poland) group (*n* = 63) receiving the drug in a dose 20 mg/kg b.w./per day (gavage feeding) for 5 days. Then, animals underwent a stroke induction procedure or sham operation. The rats were sacrificed at different time points: 3, 6, 16, or 24 h after stroke. Control groups were sham rats administered with saline solution (sham_veh_) and sham rats that received simvastatin (sham_st_). No adverse effects were observed in rats after ST administration. The study design is presented in Fig. 1.

### Permanent Middle Cerebral Artery Occlusion (pMCAO)

Stroke was induced by pMCAO using the intraluminal filament technique, as previously described [46]. Briefly, rats were anesthetized by inhalation of 2.5 vol% sevoflurane (Abbot, UK) and mixture of air and oxygen (FiO_2_ = 50%) in an induction chamber (Fig. S1, B) using *Sigma Elite Vaporizer* (Penlon, UK) (Fig. S1, C). Anesthesia was maintained by sevoflurane which end-tidal concentration was 2.0–2.2 vol%. The ventilation efficiency and the end-tidal sevoflurane concentration (Et_sevo_) were monitored by a *Vamos* analyzer (Dräger, Germany) (Fig. S1, D). Body temperature was maintained at 36–38 °C using *FST thermoregulatory system* (Fine Science Tools, USA) equipped with a thermostat, a heated pad, and a rectal thermometer (Fig. S1, A). The detailed description of all parameters monitored during and after the pMCAO procedure and sham operation is provided in the Supplementary information (Fig. S1). We did not record any significant differences in any of the registered parameters among the experimental groups (Table 1). Local anesthesia (0.5% xylocaine injection, AstraZeneca, Sweden) was administered to the anterior cervical region of the rat. Proximal fragments of the left common carotid artery (CCA), external carotid artery (ECA) and internal carotid artery (ICA) were exposed. After ligation and cutting the ECA, a nylon surgical monofilament 4–0 suture (Ethicon, UK) was inserted via its proximal end to the ICA until it blocked the origin of the MCA (approx. 17 mm). After securing the monofilament against sliding out of the vessel and homeostasis control, the wound was closed and the rat was allowed to wake up in a cage with ad libitum access to water and soft food.

**Table 1:** Physiological parameters monitored during and after pMCAO or sham surgery

Experimental Group										
Parameter	sham_VEH_	3h pMCAO	6h pMCAO	16h pMCAO	24h pMCAO	sham_ST_	3h pMCAO+ST	6h pMCAO+ST	16h pMCAO+ST	24h pMCAO+ST
Body weight (g)	376,3± 57,5	350,7 ± 37,5	386,8 ± 31,9	362,9 ± 40,2	396,6 ± 36,3	351,2 ± 37,9	348,4 ± 35,1	381,5 ± 22,6	347,7 ± 38,7	329,7 ± 4,0
Temp. (°C)	37,2 ± 0,5	37,2 ± 0,6	36,8 ± 0,4	37,0 ± 0,3	36,8 ± 0,4	37,0 ± 0,2	37,0 ± 0,2	37,7 ± 0,5	37,2 ± 0,5	37,1 ± 0,1
Et_sevo_ (vol%)	2,1 ± 0,1	2,1 ± 0,1	2,2 ± 0,09	2,1 ± 0,2	2,0 ± 0,1	2,1 ± 0,09	2,1 ± 0,08	2,1 ± 0,1	2,1 ± 0,05	2,0 ± 0,07
EtCO_2 (mmHg)_	39,0 ± 0,01	38,6 ± 0,8	38,8 ± 0,4	38,8 ± 0,4	39,1 ± 0,7	38,6 ± 1,1	38,6 ± 0,8	38,6 ± 0,8	39,2 ± 0,4	39,0 ± 0,01
SpO_2_ (%)	96,6 ± 0,8	96,8 ± 1,1	96,2 ± 0,4	96,8 ± 1,1	98,1 ± 0,7	95,8 ± 0,4	96,6 ± 0,8	96,8 ± 0,8	96,6 ± 0,8	95,6 ± 0,5
MABP (mmHg)	88,6 ± 0,5	87,8 ± 0,8	88,6 ± 1,1	87,4 ± 1,5	87,6 ± 1,7	86,2 ± 1,6	86,6 ± 0,8	87,0 ± 1,4	85,8 ± 4,1	88,0 ± 1,4
HR (l/min)	193,2 ± 7,1	188,0 ± 3,8	193,0 ± 6,4	189,2 ± 3,6	187,1 ± 5,3	185,4 ± 4,6	189,6 ± 2,4	197,2 ± 5,8	194,4 ± 6,5	189,5 ± 2,7
Glocuse (mg%)	147,4 ± 2,8	143,2 ± 4,6	144,2 ± 5,2	145,0 ± 4,3	136,5 ± 3,2	142,4 ± 4,6	145,2 ± 8,2	140,2 ± 7,3	142,2 ± 3,9	139,0 ± 2,8
Hb (mg%)	15,1 ± 0,4	14,3 ± 0,6	14,3 ± 1,09	14,4 ± 0,8	14,4 ± 0,5	14,3 ± 0,7	14,2 ± 0,4	14,5 ± 0,5	14,2 ± 0,6	14,6 ± 0,4

*Et*_*sevo*_ end-tidal concentration of sevoflurane; *EtCO*_2_ end-tidal concentration of CO_2_; *Hb* blood hemoglobin concentration; *HR* heart rate; *MABP* mean arterial blood pressure; *SpO*_2_ hemoglobin oxygen saturation. Data is expressed as mean ± SD, Shapiro-Wilk normality test followed by Bonferoni and Kruskal-Wallis tests were applied, *p* < 0.05 was considered statistically significant

### Motor Function Assessment

To assess the motor functions after stroke, we used a Bederson scale [47] with minor modifications. The rats subjected to pMCAO were graded from 1 to 3, depending on the severity of the post-stoke functional deficits. Animals presenting the flexed forelimb contralateral to the injured hemisphere (right) were graded 1 (mild functional deficit). Rats with consistently reduced resistance to lateral push toward the paretic side were graded 2 (moderate functional deficit). Rats that presented grade 1 and 2 features and circled towards the paretic side were graded 3 (severe functional deficit). We pulled the rats graded with 1 and 2 and compared them with rats presenting the severe impairment (grade 3). The motor function assessment was performed twice: 1 h following the pMCAO and 10 min. before sacrifice.

### 2, 3, 5-Triphenyltetrazolium Chloride Staining

To assess the volume of the stroke-induced cerebral infarction, we stained freshly dissected 2-mm-thick brain sections with 1.5% solution of 2, 3, 5-triphenyltetrazolium chloride (TTC, Sigma-Aldrich Co., USA) [48]. In the healthy tissue, TTC is reduced by the mitochondrial enzyme succinate dehydrogenase to a light-sensitive formazan that turns normal tissue red [49, 50]. Because in the damaged tissue this reaction cannot occur, the area remains white. After rinsing in PBS, sections were fixed in 4% formaldehyde solution, documented (Nikon D300, Japan), and analyzed. A volume of the stroke-induced infarction was estimated using the Cavalieri method [51]. Series of parallel sections containing cerebral infarction at a fixed distance from each other were analyzed using the CAST Grid analysis system. A randomly translated point grid of known area associated with each point was imposed on the studied sections. Then, the volume of infarct was calculated according to the proper mathematical formula.

### Immunofluorescence

The rats were deeply anesthetized with pentobarbital sodium (Nembutal, Lundbeck, Denmark; dose 80 mg/kg b.w., *i.p.*) and transcardially perfused with 0.9% NaCl containing heparin (5000 u, Polfa Warszawa SA, Poland), followed by 4% ice-cold paraformaldehyde in 0.01 M PBS. The brains were then removed from sculls and, after 2-h post-fixation, submersed successively in PBS with 15% and 30% sucrose until they sank. The brains were cut into 35-μm-thick coronal sections using Jung CM1800 cryostat (Leica, Germany). Immunofluorescence stainings were performed on free floating sections. The following primary antibodies were used: rabbit anti-NFκB/p65 (C-20) (1: 250 dilution; #sc-372; Santa Cruz Biotechnology, USA), rabbit anti-c-Rel (C) (1: 250 dilution; #sc-71; Santa Cruz Biotechnology), mouse anti-NeuN (1: 500 dilution; #MAB377; Merck Millipore, Germany), mouse anti-GFAP (C-GA5) (1: 400 dilution; #MAB360; Sigma-Aldrich/Merck Millipore), and mouse anti-CD11b/OX-42 (1: 50 dilution; #MCA275G; Bio-Rad Laboratories, USA). Sections were incubated with primary antibodies in PBS containing 5% normal goat serum (NGS) (JacksonImmunoResearch Laboratories, USA) and 0.25% Triton X-100 (Sigma-Aldrich) at 4 °C overnight. Primary antibodies were detected by goat anti-mouse Alexa488-conjugated (dilution 1: 500, #A-10680; ThermoFisher Scientific, USA) or goat anti-rabbit Cyanine 3-conjugated (dilution 1: 600, #A10520; ThermoFisher Scientific) secondary antibodies (1: 200 dilution). Sections were incubated with secondary antibodies at room temperature for 2 h in PBS containing 5% NGS and 0.25% Triton X-100. Finally, they were mounted on glass slides, embedded in Kaiser’s glycerol gelatin (Sigma-Aldrich), and cover-slipped. Single- and double-labelled cells were visualized using *Eclipse E600* fluorescent microscope (Nikon, Japan), documented using a confocal laser scanning microscopy system LSM 700 (Zeiss, Jena, Germany) using a 20 × 0.8 plan apochromat objective lens, and analyzed using *ZEN 2009* software (Zeiss). Channels were scanned consecutively to avoid cross-talk.

### Western Blotting

Freshly extracted brain tissue was used for the separate extraction of cytoplasmic and nuclear proteins with the *NE-PER kit* (Thermo-Fisher Scientific, USA), according to the manufacturer’s instructions. The total protein concentration of each protein fraction was determined using the *Pierce BCA Protein Assay Kit* (Thermo-Fisher Scientific), according to the manufacturer’s protocol. Samples containing 30 μg of the extract were then mixed with reducing SDS/PAGE sample buffer and incubated at 94 °C for 5 min. before performing SDS/PAGE electrophoresis. Afterwards, proteins were transferred (Mini Trans-Blot Cell, Bio-Rad Laboratories) onto nitrocellulose membrane (0.2 μm, Bio-Rad Laboratories). Immunoblots were incubated with primary antibodies against the NFκ-B/p65 (C-20) (1: 500; #sc-372; Santa Cruz Biotechnology) or c-Rel (B-6) (1: 100; #sc-6955; Santa Cruz Biotechnology) in 1.5% non-fat milk at 18 °C overnight. After rinsing in T-PBS, blots were incubated with the goat anti-rabbit (1: 5500; #31,460; Invitrogen/ThermoFisher Scientific) or the rabbit anti-mouse (1: 5000; #634,426; Sigma-Aldrich/Millipore) HRP-conjugated secondary antibodies for 2 h in RT. The blots with immuno-reactive bands were developed using *SuperSignal West Pico* (ThermoFisher Scientific), exposed to X-ray film (*CL-XPosure Film*, ThermoFisher Scientific) for 2–3 min., documented with HP Scanjet G3110 scanner, and analyzed with ImageJ 1.46 software (NIH, USA). After imaging, in order to verify the equal protein loading, the immunoblots were also incubated with an antibody against β-actin (1: 3000; #A5441; Sigma-Aldrich). Three to five different sets of experiments were performed for each time point in the pMCAO and pMCAO + ST groups.

### Electrophoretic Mobility Shift Assay

To study NFκ-B-DNA binding activity, we used *LightShift Chemiluminescent EMSA Kit* (ThermoFisher Scientific), according to the manufacturer’s instructions. The sequences of the two biotin 3′end-labeled ssDNA (IDT, USA) used in the hybridization reaction are shown in Table S1 (Supplementary information). The optimal conditions for the protein–DNA binding reaction were selected experimentally (Fig. S3 in Supplementary information). The biotin end-labeled dsDNA was incubated with a nuclear extract for 20 min. in RT followed by 10 min. in 37 °C, and then electrophoresed (*MiniProtean 3*, Bio-Rad Laboratories) on 5% native gel. The DNA was then rapidly (30 min.) transferred to a positive nylon membrane (Zeta-Probe, 0.45 μm, Bio-Rad Laboratories), UV crosslinked (UV light: 254 mm, 120 mJ/cm^2^, 60 s.), probed with streptavidin-HRP conjugate, and incubated with the substrate (*Chemiluminescent Nucleic Acid Detection Module*, ThermoFisher Scientific). Immuno-reactive bands were exposed to X-ray film (*CL-XPosure Film*, ThermoFisher Scientific), documented with HP Scanjet G3110 scanner, and analyzed with ImageJ 1.46 software (NIH). Three to five different sets of experiments were performed for each time-point in the pMCAO and pMCAO + ST groups. The NFκ-B-DNA binding activity was analyzed based on comparison of the optical density (OD) in the pMCAO and pMCAO + ST groups with OD in the control groups (sham_veh_ and sham_st_, respectively) and expressed as % of control.

### Quantitative RT-PCR

To quantify mRNA level of the genes regulated by NFκ-B transcription factor, total RNA was extracted using *Total RNA Mini* kit (A&A Biotechnology, Poland), according to the manufacturer’s protocol. To eliminate possible DNA contamination, the RNA was treated with DNase I (Promega Co, USA). Total mRNA was reverse transcribed into cDNA by M-MLV reverse transcriptase (Promega Co., USA) in *iCycler* (Bio-Rad Laboratories). Five out of six qPCR amplification sets were designed de novo. To design primers for *Noxa*, *Bcl-2*, *Bcl-x*, *Actb* and *Gapdh*, we used BLAST (http://blast.ncbi.nlm.nih.gov/Blast.cgi). The generated by Primer-BLAST sequences were analyzed by Vector NTI Advance (LifeTechnologies, USA) and Dr. Zuker’s mfold (USA). After confirming the specificity, we ordered the primers’ synthesis (IDT, USA; Blirt and Genomed, Poland). Primer sequences for *Puma* were available in the literature [52]. The expression levels of mRNAs were measured by SYBR green based quantitative RT-PCR (qRT-PCR; Roche Applied Science, USA). Samples (*n* = 6 rats per group) were run in triplicates. qRT-PCR reaction conditions and primer sequences are provided in Table S2 (Supplementary information). As reference genes, based on the literature search, we selected two basic metabolism genes: *Actb* (β-actin) and *Gapdh* (glyceraldehyde 3-phosphate dehydrogenase). However, due to the detected differences in *Gapdh* expression level between the sham and pMCAO groups, we excluded this gene from the analysis. *Actb* mRNA expression level was affected neither by stroke nor simvastatin treatment, and we used this gene as the internal reaction control in our study. To estimate the expression level of the target genes, we used the basic method of relative quantification with a calibrator (i.e., a sample of healthy brain tissue with a constant ratio of number of transcripts of a target gene to a reference gene). This method is based on ΔΔ*C*_T_-method [53], where the threshold cycle (*C*_T_) was calculated by *LightCycler 480* software (Roche Applied Science).

### Statistical Analysis

In our study, no sample calculation was performed. All data were checked by the Shapiro–Wilk and Kolmogorov–Smirnov normality tests and analyzed as follows:

1. to assess potential differences among the experimental groups in physiological parameters monitored during/after pMCAO procedure and sham surgeries, Bonferroni and Kruskal–Wallis tests were applied
2. to compare the size of cerebral infarct between the 24 h pMCAO and 24 h pMCAO + ST groups, unpaired, two-tailed *t* test was applied
3. to compare motor function impairment between the pMCAO and the pMCAO + ST groups, Fisher’s exact test was applied
4. to compare NF-κB-DNA binding activity and gene expression level between sham_VEH_ and each of the pMCAO groups and between sham_ST_ and each of the pMCAO + ST groups, ordinary one-way ANOVA followed by the two-stage step-up method of Benjamini, Krieger, and Yekutieli was applied
5. to compare gene expression levels and protein levels between the pMCAO and the pMCAO + ST groups at different time points, two-way ANOVA followed by the two-stage step-up method of Benjamini, Krieger, and Yekutieli was applied

Data was analyzed by GraphPad Prism 8 (USA) and STATISTICA 13.3 (USA) and is presented as bar graphs showing mean ± SD. Differences between the groups were considered significant when *p* values were less than 0.05.

## Results

### Simvastatin Reduces Infarct Volume and Improves Neurological Function in pMCAO Rats

**Fig. 2:**
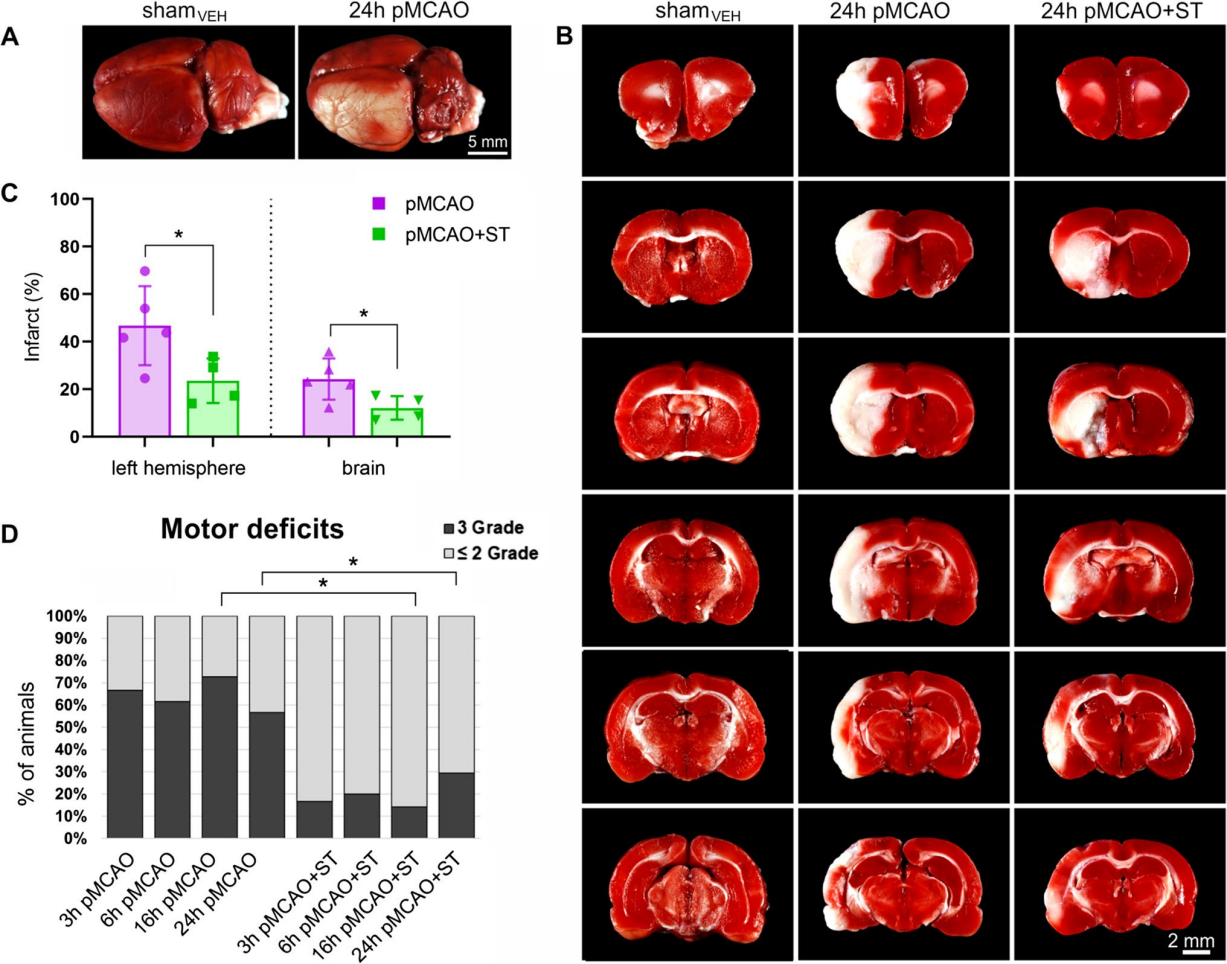
Short-term simvastatin pretreatment reduces cerebral infarct and improves neurological functions. Representative images of the control (sham_VEH_) brain and rat’s brain 24 h after pMCAO with the infarct area (white) visible within the left hemisphere (**A**), and rat brain sections from the pMCAO group, pMCAO group treated with simvastatin (ST), and the control group stained with 1.5% 2,3,5-triphenyltetrazolium chloride (TTC) (**B**). Percentage of infarct volume in the left hemisphere and the whole brain 24 h after pMCAO (**C**). Motor deficits assessed 1 h after pMCAO in rats with different survival periods and in pMCAO rats pretreated with simvastatin for 5 days before stroke (**D**). Grade ≤ 2 indicates mild and moderate brain damage symptoms, and Grade 3 indicates severe brain damage symptoms. Two-tailed *t*-test (in C) and exact Fisher’s test (in D). Histograms show mean ± SD, ^*^p < 0.05

The results show that 24 h after pMCAO the infarct area comprised 46.7 ± 16.6% of the ipsilateral hemisphere and 24.3 ± 9.4% of the whole brain (Fig. 2A–C). Whereas in rats pretreated with ST prior to stroke, the infarction comprised 23.6 ± 8.7% and 12.1 ± 5.0% of the ipsilateral hemisphere and the total brain, respectively (Fig. 2A–C). The evolution of the cerebral infarct up to 24 h is presented in Fig. S2 (Supplementary information). The morphological changes induced by ST treatment were accompanied by a reduction in the stroke-induced functional deficits. Severe neurological deficits (Grade 3 on the Bederson scale) were observed in 66%, 61%, 73%, and 57% of the rats at 3, 6, 16, and 24 h after pMCAO, respectively (Fig. 2D). At all the study time points, the stroke rats pretreated with ST presented significant improvement of the neurological score as assessed by the Bederson scale. The percentage of the ST-treated rats with severe neurological deficits was 17, 20, 15, and 29 in the consecutive hours after pMCAO (Fig. 2D). Consequently, 49%, 41%, 58%, and 28% more rats from the ST-treated groups showed less severe (Grade 2), instead of more severe, neurological deficits (Grade 3) compared with the stroke controls, at 3, 6, 16, and 24 h after pMCAO, respectively.

### Simvastatin Reduces RelA and Transiently Increases c-Rel in the Nuclear Fraction of pMCAO Rats

**Fig. 3:**
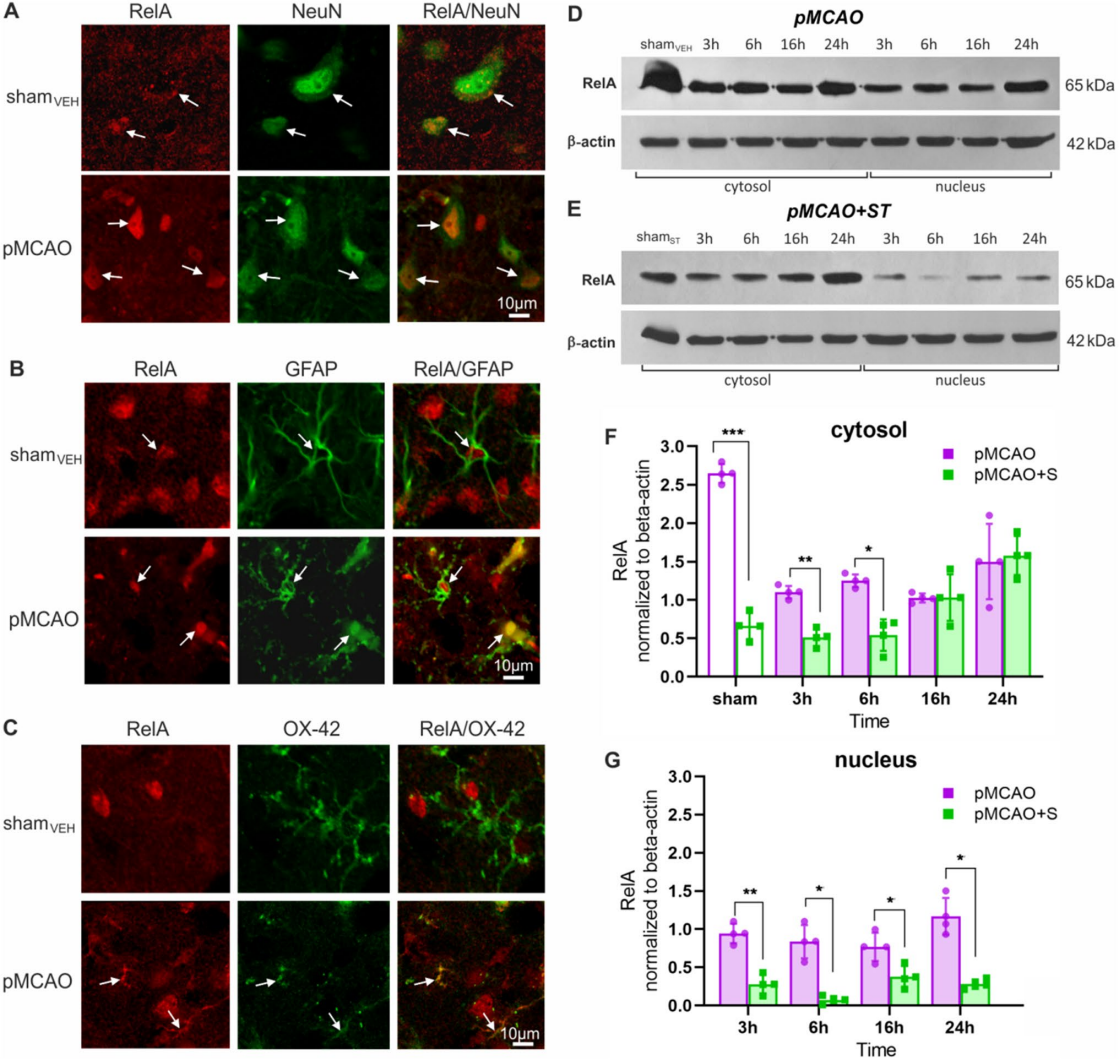
Stroke induces nuclear translocation of the NF-κB RelA subunit in neurons 3 h after pMCAO and pretreatment with simvastatin inhibits RelA translocation. Representative confocal scanning microscope images of RelA/NeuN + (**A**), RelA/GFAP + (**B**), and RelA/OX-42 + (**C**) cells in the control (sham_VEH_) and ipsilateral hemispheres of the pMCAO rats. Representative bands showing RelA level in sham_VEH_ group and at 3, 6, 16, and 24 h after pMCAO normalized against β-actin (**D**), as well as in the sham_ST_ group and ST-treated rats at 3, 6, 16, and 24 h after pMCAO normalized against β-actin (**E**). Relative RelA protein levels in cytosolic (**F**) and nuclear fractions (**G**). Two-way ANOVA followed by the two-stage step-up method of Benjamini, Krieger, and Yekutieli. Histograms show mean ± SD; ^*^p < 0.05, ^**^p < 0.01, ^***^p < 0.001

In the control group, the RelA subunit of NF-κB factor was detected in neurons and GFAP + astrocytes present in the striatum and cortex (Fig. 3A and B). In most neurons, RelA was in the cytoplasm, while in astrocytes, this subunit was present only in the nucleus (Fig. 3B). After stroke, we observed translocation of RelA from the cytoplasm to the nucleus in neurons within the peri-infarct area (Fig. 3A). This occurred as early as 3 h after pMCAO (data not shown). At 24 h after pMCAO, in the peri-infarct area, we observed scattered microglial cells with RelA present in the nucleus (Fig. 3C). Western blot results confirmed the presence of RelA in both the cytoplasm and the nucleus after pMCAO (Fig. 3D and F–G). However, we did not detect RelA in the nuclear fraction from the control group (sham_VEH_) (data not shown). In the group treated with ST, we observed a decrease in RelA in both the cytosolic and nuclear fractions compared with the sham_ST_ rats (Fig. 3E–G).

**Fig. 4:**
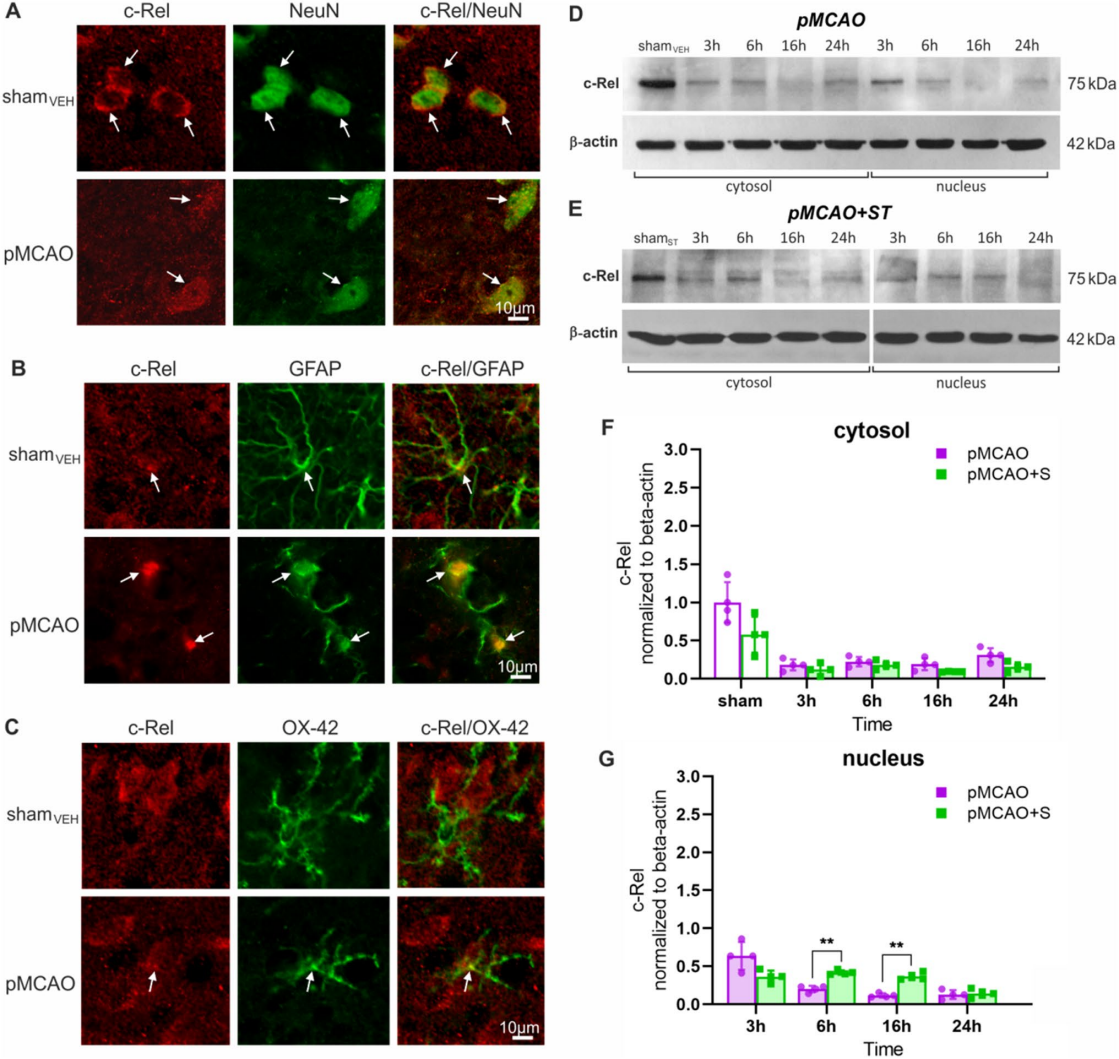
Stroke induces nuclear translocation of the NF-κB c-Rel subunit in neurons 3 h after pMCAO, and pretreatment with simvastatin induces a transient c-Rel translocation. Representative confocal scanning microscope images of c-Rel/NeuN + (**A**), c-Rel/GFAP + (**B**), and c-Rel/OX-42 + (**C**) cells in sham_VEH_ and ipsilateral hemisphere of the pMCAO rats. Representative bands showing c-Rel level in the sham_VEH_ group and at 3, 6, 16, and 24 h after pMCAO normalized against β-actin (**D**), as well as in the sham_ST_ group and ST-treated rats at 3, 6, 16, and 24 h after pMCAO normalized against β-actin (**E**). Relative c-Rel protein levels in cytosolic (**F**) and nuclear fractions (**G**). Two-way ANOVA followed by the two-stage step-up method of Benjamini, Krieger, and Yekutieli. Histograms show mean ± SD, ^**^p < 0.01

The NF-κB c-Rel subunit was present in neurons and GFAP + astrocytes in both the striatum and cortex of sham_VEH_ rats (Fig. 4A and B). In neurons, this subunit was present in the cytoplasm, while in astrocytes, it was in the nucleus. We did not detect c-Rel + microglia in the brains of rats from the control group (Fig. 4C). After pMCAO, we observed nuclear translocation of c-Rel in neurons localized in the peri-infarct area (Fig. 4A). Additionally, we detected c-Rel in the nuclei of microglial cells in the stroke group (Fig. 4C). Western blot results showed expression of c-Rel in the cytosolic fraction of the control group (Fig. 4D). However, we did not detect this subunit in the nuclear fraction. In the consecutive hours after pMCAO, the level of c-Rel in the cytosol was decreased compared with the control group (Fig. 4D and F). A low concentration of c-Rel was also present in the nuclear fraction. In the rats treated with ST, we recorded no significant changes in c-Rel level in the cytosolic fraction compared with the untreated-rats with pMCAO (Fig. 4E and F**)**. However, at 6 and 16 h, we detected a slightly higher c-Rel expression in the nuclear fractions of ST-treated compared with untreated stroke rats (Fig. 4E and G).

### Simvastatin Modifies NF-κB Binding Activity in the Infarct and Peri-infarct Areas After pMCAO

**Fig. 5:**
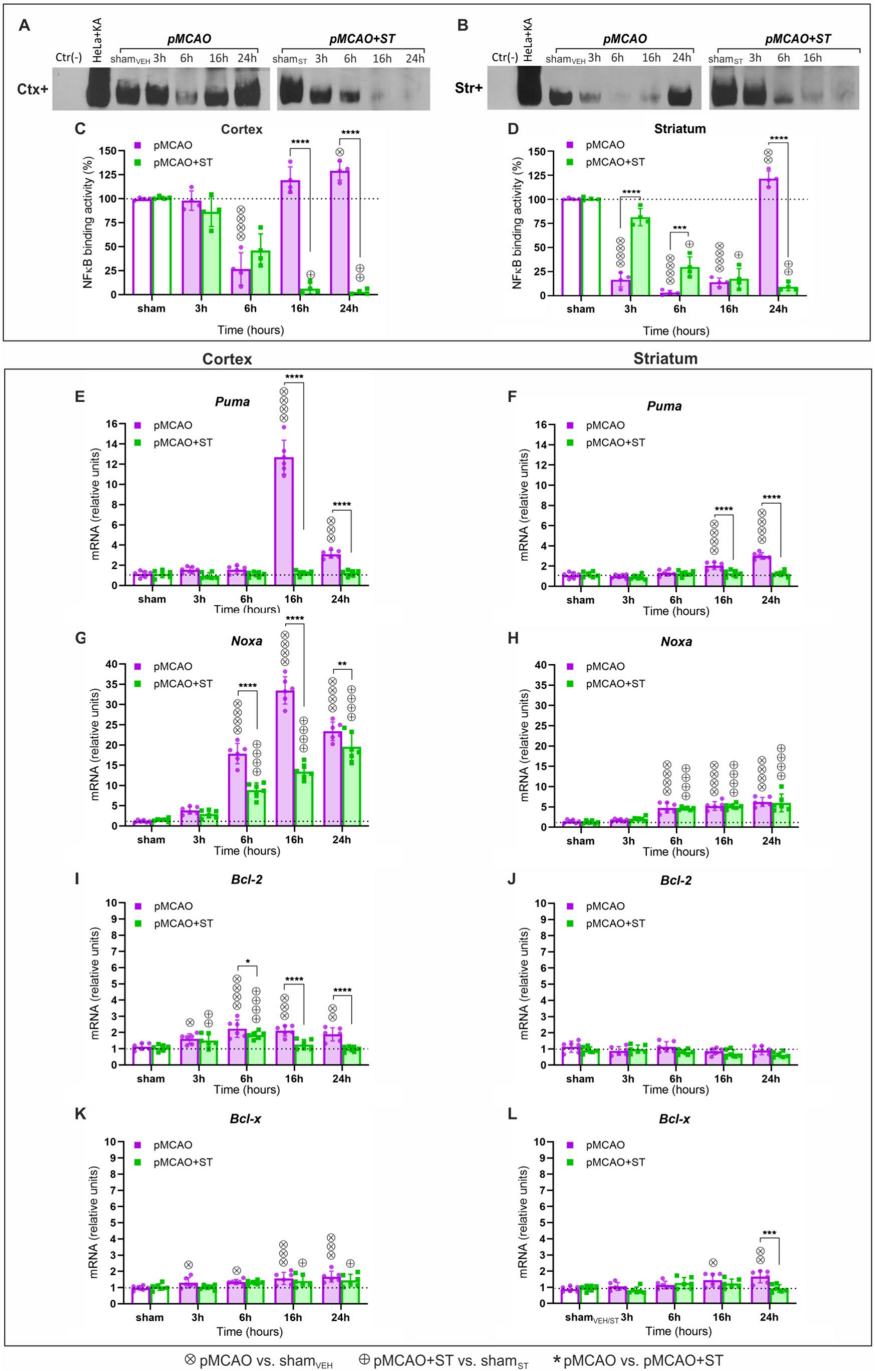
Simvastatin affects the DNA binding activity of NF-κB and inhibits the expression of the stroke-induced genes regulated by this transcription complex. Representative bands showing binding of NF-κB to κB consensus sequence in the sham rats, in the pMCAO rats, and in the pMCAO rats pretreated with simvastatin (ST) at 3, 6, 16, and 24 h following stroke in the cortex (Ctx +) (**A**) and striatum (Str +) (**B**). The NF-κB-DNA binding activity in the pMCAO and pMCAO + ST groups in the cortex (**C**) and striatum (**D**). Protein–DNA binding activity was analyzed by EMSA. Data represent the percent of sham_VEH_ (for pMCAO) and sham_ST_ (for pMCAO + ST). *Puma* expression in the sham, pMCAO, and pMCAO + ST groups in the cortex (**E**) and striatum (**F**). *Noxa* expression in sham, pMCAO, and pMCAO + ST groups in the cortex (**G**) and striatum (**H**). *Bcl-2* expression in sham, pMCAO, and pMCAO + ST groups in the cortex (**I**) and striatum (**J**). *Bcl-x* expression in sham, pMCAO, and pMCAO + ST groups in the cortex (**K**) and striatum (**L**). Gene expression was measured by qRT-PCR and normalized to *Actb* mRNA expression. Ordinary one-way ANOVA (sham_VEH_ vs. pMCAO, and sham_ST_ vs. pMCAO + ST group comparisons) and two-way ANOVA (pMCAO vs. pMCAO + ST group comparisons) followed by the two-stage step-up method of Benjamini, Krieger, and Yekutieli. Histograms show mean ± SD, ^*^, ^⊗^, ^⊕^*p* < 0.05, ^**^, ^⊗⊗^, ^⊕⊕^*p* < 0.01, ^***^, ^⊗⊗⊗^, ^⊕⊕⊕^*p* < 0.001, and ^****^, ^⊗⊗⊗⊗^, ^⊕⊕⊕⊕^*p* < 0.0001

The results showed a 73% decrease in NF-κB binding activity to the consensus κB sequence in the frontoparietal cortex compared with the control group (sham_VEH_) 6 h after pMCAO (*p* < 0.0001) (Fig. 5A and C). However, after 24 h, we recorded a 29% increase in NF-κB binding activity *versus* that of the control (*p* = 0.0299). In the cortex of ST-treated rats, we recorded a gradual decrease in NF-κB binding activity compared with the vehicle group (sham_ST_), reaching 6 ± 7% and 3 ± 3% of control in the 16- and 24-h post-stroke groups (*p* = 0.0185 and *p* = 0.0098, respectively). Additionally, at 16 and 24 h after stroke, NF-κB binding activity in the cortex of the ST-treated rats significantly decreased compared with the untreated group (*p* < 0.0001 for both comparisons) (Fig. 5A and C).

In the striatum, NF-κB binding activity decreased at 3, 6, and 16 h after stroke compared with the control group (Fig. 5B and D) and accounted for 17 ± 9%, 3 ± 3%, and 14 ± 6% of the control group, respectively (*p* < 0.0001 for each comparison). However, in the 24-h post-stroke group, we found a 22% increase in NF-κB binding activity *versus* sham_VEH_ (*p* = 0.0035). In the striatum of ST-treated rats, NF-κB binding activity decreased gradually over the observation period (Fig. 5B and D) and accounted for 82 ± 11%, 30 ± 13%, 18 ± 13%, and 9 ± 5% of the sham_ST_ group, respectively. We observed an increase in NF-κB binding activity in ST-treated rats compared with untreated rats at 3 and 6 h after stroke (*p* < 0.0001 and *p* = 0.0007, respectively). There was no significant difference in NF-κB binding activity between the ST-treated rats and the untreated rats 16 h after stroke (*p* = 0.3211). In the 24-h post-stroke group, we observed a significant decrease in NF-κB binding activity in the pMCAO + ST group compared with the pMCAO group (*p* < 0.0001).

### Simvastatin Inhibits the Expression of Stroke-Induced Pro- and Anti-apoptotic Genes Regulated by NF-κB

Neither in the peri-infarct area (cortex) nor in the infarct area (striatum) changes in *Puma* expression were recorded compared with shams at 3 and 6 h after stroke (Fig. 5E and F). At 16 h after pMCAO, a significant increase in *Puma* mRNA expression was observed in the cortex (Fig. 5E). We recorded an 11.3-fold higher expression of this gene compared with the control group (*p* < 0.0001). An increase, although not as large (2.75-fold), was also present 24 h after stroke (*p* = 0.0003 compared with the sham_VEH_). The results showed a significant decrease in *Puma* expression in ST-treated pMCAO rats *versus* vehicle pMCAO rats 16 and 24 h post-stroke (*p* < 0.0001 for both comparisons). In the striatum, we recorded an increase in *Puma* expression compared with the control in the 16- and 24-h post-stroke groups (*p* < 0.0001 for both comparisons) (Fig. 5F). In ST-treated rats, *Puma* mRNA level decreased compared with the pMCAO group 16 and 24 h after stroke (*p* < 0.0001 for both comparisons).

The results showed no significant changes in *Noxa* mRNA levels in either the cortex or the striatum 3 h after the stroke (Fig. 5G and H). However, we noted a significant increase in *Noxa* expression in the cortex in the remaining stroke groups (Fig. 5G). At 6, 16, and 24 h, the mRNA level was, respectively, 15.2-, 28.5-, and 19.9-fold higher compared with sham_VEH_ (*p* < 0.0001 for each comparison). In ST-treated rats, *Noxa* levels were lower compared with pMCAO rats in the 6-, 16-, and 24-h groups (*p* < 0.0001, *p* < 0.0001, and *p* = 0.0018, respectively), but still higher than in the shams. In the striatum, *Noxa* mRNA levels in the pMCAO group were 3.4-, 3.7-, and 4.4-fold higher compared with sham_VEH_ at 6, 16, and 24 h post-stroke (*p* < 0.0001 for each comparison) (Fig. 5H). At the same time points in ST-treated rats, *Noxa* expression was also increased compared with sham_ST_ (*p* < 0.0001 for each comparison). However, we did not detect any significant differences between the ST-treated and untreated rats.

We found increased *Bcl-2* expression in the cortex of the pMCAO rats (Fig. 5I). After 3, 6, 16, and 24 h, mRNA levels were 1.4-, 2.0-, 1.9-, and 1.7-fold higher, respectively, compared with sham_VEH_ (*p* = 0.0312, *p* < 0.0001, *p* = 0.0002, and *p* = 0.0021). In the ST-treated group, we observed increased *Bcl-2* expression at 3 and 6 h (*p* = 0.0088 and *p* < 0.0001, respectively, compared with the vehicle control), but mRNA levels in the treated rats were lower compared with vehicle rats at 6, 16, and 24 h after stroke (*p* = 0.0416, *p* < 0.0001, and *p* < 0.0001, respectively). There were no significant changes in *Bcl-2* expression in the striatum in either the pMCAO or pMCAO + ST rats compared with the sham groups (Fig. 5J).

Results showed increased post-stroke *Bcl-x* mRNA expression in the frontoparietal cortex and striatum (Fig. 5K and L). In the cortex, we observed 1.3-, 1.4-, 1.6-, and 1.7-fold increases in *Bcl-x* at 3, 6, 16, and 24 h after pMCAO (*p* = 0.0158, *p* = 0.0117, *p* = 0.0007, and *p* = 0.0003, respectively) (Fig. 5K). The results also showed an increase in *Bcl-x* expression in rats treated with ST 16 and 24 h after stroke (*p* = 0.022 and *p* = 0.022, respectively). In the striatum, we found an increase in *Bcl-x* expression only at 16 and 24 h (*p* = 0.0208 and *p* = 0.0015, respectively, compared with sham_VEH_) (Fig. 5L). After 16 h, there was no significant difference in *Bcl-x* expression between the pMCAO and the pMCAO + ST rats (*p* = 0.2953). However, after 24 h, *Bcl-x* expression was lower in ST-treated rats compared with untreated rats (*p* = 0.0002).

## Discussion

In this study, we investigated the effect of HMG-CoA inhibitor simvastatin on the activation pattern of NF-κB, cytosolic and nuclear subunit content, and the expression of some pro- and anti-apoptotic genes regulated by this transcription factor in the acute stroke phase. Short-term pretreatment with simvastatin induced reduction in the cerebral infarct and the significant improvement of the motor activity in the middle-aged rats, which correlated with a significant reduction of the RelA subunit and a transient increase of c-Rel in the nuclear fraction, changes in the NF-κB-DNA binding activity, and downregulation of the NF-κB-regulated apoptotic genes.

The neuroprotective effects of statins have been well-documented in ischemic stroke models. Indeed, various statins have been used, at different dosages and with different routes of administration, in pre- or post-stroke paradigms in various stroke models (reviewed in [17]), [44, 45]. The results of the recently published meta-analysis of 24 animal studies have shown that simvastatin is one of the two most effective statins in reducing infarct volume [17]. Specifically for this statin, 40–50% reduction of the infarct volume was recorded [54–56]. In different studies, the applied statin dose ranged from 0.2 to 40 mg/kg b.w. Interestingly, Christophe and colleagues reported that the statin-mediated effect on infarct size was independent of the drug dosage and time of administration [17]. It has been, however, dependent on the drug administration route, and, surprisingly, the analysis showed that subcutaneous injection has been the most effective in animal models of stroke. However, in our study, simvastatin was administered by gavage, which better reflects the clinical use of this drug. Considering the functional outcome after stroke, HMG-CoA inhibitors improve motor and cognitive functions in rodents (reviewed in [17]), [44]. Results of our study showing 50% reduction of the infarct and significant improvement of neurological outcome in the pMCAO model after the short-term simvastatin pretreatment (5 days, 20 mg/kg b.w., gavage) are in line with previous studies. However, it should be emphasized that most of the studies on the effectiveness of statins after stroke were conducted in young adult or adult rodents, while in our study we used middle-aged rats, as recommended [57].

As a factor regulating gene expression of proteins involved in many processes (see “Introduction”), NF-κB is highly expressed in all cell populations in the CNS. Previous studies showed the opposite action of dimers containing RelA and c-Rel subunits and different roles of NF-κB depending on the cell type (summarized in [32]). In our study, in the brain tissue of naïve middle-aged rats, we observed expression of RelA and c-Rel subunits in neurons (cytosol) and astrocytes (nucleus), but not in microglia. Our results in the rat stroke model are in line with previous reports [35, 36, 58]. After pMCAO, we observed increased nuclear translocation of the RelA-containing dimers and decreased translocation of the c-Rel-containing dimers. Both subunits were present in the nuclei of neurons, astrocytes, and microglia.

In the ischemic brain tissue of rats treated with simvastatin, as expected, the level of RelA in the nucleus significantly decreased. This effect was already present very early after stroke (at 3 h). We also observed a transient increase in c-Rel at 6 and 16 h after pMCAO. It should be emphasized, however, that the content of c-Rel in the nuclear fraction was still significantly lower than in the control group. Thus, simvastatin not only blocked the nuclear translocation of RelA-containing dimers but also transiently promoted c-Rel nuclear translocation after pMCAO. A relevant question regarding the statin mechanism of action was whether its neuroprotective effect in stroke may be, at least partially, a result of not only downregulation of pro-apoptotic genes regulated by RelA-containing dimers but also of upregulation of anti-apoptotic genes regulated by c-Rel-containing dimers of NF-κB. To answer this question, we investigated the DNA binding activity of this transcription factor and NF-κB-regulated apoptotic gene expression. The binding activity of the NF-κB transcription complex (mainly RelA and p50 subunits) with DNA at consensus sequence present within promotor regions of the transcriptionally regulated genes was previously investigated in focal and global cerebral ischemia models. Gabriel et al. found no significant changes in NF-κB-DNA binding activity up to 24 h post-ischemia after 50 min MCAO/reperfusion [59]. In models with a longer vessel occlusion period (90–120 min), an increased NF-κB-DNA binding activity was observed after 20 h and 5 days after ischemia/reperfusion [60, 61]. In the 4-vessel occlusion model followed by reperfusion after 30 min, the increased DNA binding activity of NF-κB was observed after 24 and 72 h [62, 63]. In the pMCAO model, we observed a transient decrease in NF-κB binding activity early on (3 and 6 h), followed by an increase at 16 h and 24 h (frontoparietal cortex) and 24 h (striatum). The lack of changes in the binding activity in the early hours after transient ischemia could be explained by substantial neuronal cell death and delayed activation of NF-κB in reactive glia with accompanying de novo synthesis of RelA [59]. In a more invasive model with permanent occlusion of MCA, used in our study, the glia activation is more extensive and occurs sooner [64, 65]; this can explain the earlier increase of the NF-κB transcriptional activity.

In our study, simvastatin decreased the stroke-induced NF-κB-DNA binding activity observed in the late hours of the first day (at 16 and 24 h in the cortex and at 24 h in the striatum). In the pMCAO + ST group, we also recorded the increased DNA binding activity of this factor in early hours (3 and 6 h) compared with the untreated rats with pMCAO. This effect was present only in the striatum, where stroke triggers rapid cell death and NF-κB binding activity is extremely low during that time. The increase in NF-κB binding activity in the statin-treated animals can be explained by a greater number of surviving cells (smaller infarct volume). Therefore, simvastatin shows a normalizing effect on the NF-κB-DNA binding activity after stroke.

*Puma* and *Noxa* are two pro-apoptotic genes whose expression is regulated by NF-κB dimers containing RelA/p65 subunit. Although previous reports suggested that *Puma* was not expressed in the adult brain under physiological conditions [66], our results showed a basic expression level of this gene in the frontoparietal cortex and striatum of naïve rats. Previous studies have shown upregulation of *Puma* during the first 8 h and 4–10 days after transient MCAO [67, 68]. We recorded an increase in *Puma* expression in the cortex and striatum earlier, at 16 and 24 h after pMCAO. Maximum expression was recorded at 16 h in the striatum. Interestingly, studies on transgenic mice *Puma*^*−/−*^ showed that a lack of this protein had no influence on the size of ischemic infarct and neurological deficit after transient MCAO [67]. This suggests that it either does not contribute significantly to lesion development or that there are some compensatory mechanisms and that the role of this protein in stroke should be considered in the context of other proteins from “BH3-only” protein (BOP) family. The results of our study showed a stroke-induced increase in *Noxa* expression in the cortex and striatum. The elevated mRNA level of this gene was already present at 6 h after pMCAO. Again, the expression of *Noxa* in the cortex was significantly higher than in the striatum. On the one hand, this should be expected given the excessive cell loss during the first post-stroke hours due to necrosis in the ischemic core, which occupied most of the striatum in our ischemic model. On the other hand, the substantial *Noxa* increase in the cortex (reaching a peak with a 28-fold increase compared with the control) suggests a more important role of this protein in stroke-induced apoptosis than previously thought.

We showed that short-term pretreatment with simvastatin completely inhibited the stroke-induced increase in *Puma* expression (in both the cortex and striatum) and partially inhibited the elevated *Noxa* expression (only in the cortex). Considering that simvastatin is an unspecific NF-κB inhibitor, it could also block other transcription factors regulating *Puma* expression, such as p53. Since the *p53* gene is also regulated by NF-κB, it is possible that NF-κB regulates *Puma* transcription not directly, but via p53 [69, 70]. However, p53-independent regulation of *Puma* has also been reported [71]. The fact that simvastatin did not completely inhibit *Noxa* expression suggests the presence of additional NF-κB-independent regulation mechanisms of this gene in the ischemic brain. Indeed, transcription of *Noxa* could also be regulated by p53 family proteins, such as p53 itself, p63, and p73 [52, 72].

NF-κB dimers containing the c-Rel subunit regulate transcription of *Bcl-2* and *Bcl-x* genes. BCL-2 protein, a precursor of all proteins from its family, plays an important role in maintaining the mitochondrial integrity necessary to keep cells alive under ischemic conditions [73]. Activation of *Bcl-2* was investigated in a few models of ischemic stroke [35, 74–76], and the results varied depending on the brain area investigated and the intensity of the ischemic process. Some studies showed no change in *Bcl-2* [35], while in others, an increased [74] or a decreased [75] expression was observed. In the focal thrombosis model, Isenmann et al. showed an increase in BCL-2 levels in the peri-infarct area but a decrease in the infarct area [76]. In the pMCAO model, we recorded a slight increase in *Bcl-2* (reaching a peak with a twofold increase compared with the control at 6 h) in the cortex and no changes in its expression in the striatum. Another protein determining fate of CNS cells under ischemic conditions is BCL-X_L_ [77]. Expression of the *Bcl-x* gene was investigated in various ischemic models. Most studies reported rapid but transient increases in *Bcl-x* mRNA levels in the cortex and hippocampus [74, 77–79]. After 24 h, *Bcl-x* expression returned to baseline, accompanied by an increase in apoptosis. We found a slight increase in *Bcl-x* expression in the cortex and striatum after stroke. Observed in our study, the low level of c-Rel in the nuclear fraction may explain the low transcription of anti-apoptotic genes regulated by dimers of NF-κB containing this subunit. Moreover, ischemic conditions induce alternative mRNA splicing, and instead of anti-apoptotic BCL-XL, a pro-apoptotic BCL-XS protein can be synthesized [76].

In our study, 5-day simvastatin treatment in a dose of 20 mg/kg b.w. decreased *Bcl-2* expression in the cortex and *Bcl-x* expression in the striatum of pMCAO rats. Therefore, simvastatin did not induce the NF-κB-mediated anti-apoptotic mechanisms in the early post-stroke phase. Chen et al. reported that statins administered orally for 7 days in very low doses (1 mg/kg of atorvastatin and 3 mg/kg b.w. of simvastatin), starting 24 h after transient MCAO, did not reduce the infarct area but improved the neurological outcome in rats, which was accompanied by induction of angiogenesis, synaptogenesis, and neurogenesis [80]. These data suggest that apart from the neuroprotective action of HMG-CoA inhibitors in acute stroke, they may also participate in the recovery processes in the prolonged stroke phase. Considering that most of the research done so far has focused on the early post-stroke time (hours and days), this may be an interesting topic for further research.

## Conclusions

In summary, this study highlights two important points. First, it indicates the multidirectional involvement of NF-ĸB transcription factor in both neurodegenerative and neuroprotective processes in the course of ischemic stroke. Second, it shows a clear association between the prophylactic administration of simvastatin and the modification of the NF-ĸB pathway that underlies its neuroprotective effect in acute stroke. The results show that short-term pretreatment with simvastatin normalizes the DNA binding activity of NF-κB and inhibits the expression of pro- and anti-apoptotic genes regulated by various subunits of this transcription factor in a permanent MCAO model. Our study provides new insights into statin action in acute stroke, which may be helpful in designing more selective pharmacological strategies against stroke, based on the modulation of the NF-κB pathway, with an opportunity to obtain better therapeutic outcomes. Finally, this study contributes to the discussion on the prophylactic use of statins in patients at high risk of stroke.

## Supplementary Information

Below is the link to the electronic supplementary material.

Supplementary file1 (PDF 541 KB)

## Data Availability

The datasets generated during and/or analyzed during the current study are available from the corresponding author on reasonable request.
