# Supplementary material for: The Effect of Simvastatin on the Dynamics of NF-κB-Regulated Neurodegenerative and Neuroprotective Processes in the Acute Phase of Ischemic Stroke
Source: Mol Neurobiol. 2023 May 19;60(9):4935–51. doi: 10.1007/s12035-023-03371-2 (PMC10415422; doi:10.1007/s12035-023-03371-2)
Supplement: Supplementary file 1 — Supplementary file1 (PDF 541 KB) [file 12035_2023_3371_MOESM1_ESM.pdf]

Supplementary Information to:

The effect of simvastatin on the dynamics of NF-κB-regulated neurodegenerative and neuroprotective processes in the acute phase of ischemic stroke, *Molecular Neurobiology*

Grazyna Lietzau[1*], Waldemar Sienkiewicz[2], Zbigniew Karwacki[3†], Jerzy Dziewiątkowski[1], Jerzy Kaleczyc[2], Przemysław Kowiański[1,4]

[1]Division of Anatomy and Neurobiology, Faculty of Medicine, Medical University of Gdańsk, Dębinki 1, 80-211 Gdańsk, Poland; [2]Department of Animal Anatomy, Faculty of Veterinary Medicine, University of Warmia and Mazury, Oczapowskiego 13, 10-719 Olsztyn, Poland; [3]Department of Neuroanaesthesiology, Faculty of Medicine, Medical University of Gdańsk, Dębinki 7, 80-211 Gdańsk Poland; [4]Institute of Health Sciences, Pomeranian University in Słupsk, Bohaterów Westerplatte 64, 76-200 Słupsk, Poland

Corresponding author: Grazyna Lietzau (Ph.D.): grazyna.lietzau@gumed.edu.pl

Physiological parameters monitored during and after permanent Middle Cerebral Artery occlusion (pMCAO) procedure and sham operation

Each rat was weighed prior to induction of stroke (**Table 1**). The weight was also monitored after pMCAO. During the procedure, mean arterial blood pressure (MABP), heart rate (HR), end-tidal concentration of CO_2 ($EtCO_2$) and sevoflurane (Et_{sevo}), as well as internal body temperature were monitored and kept within a physiological range. Particular attention has been paid to the value of these parameters when introducing nylon suture into ICA and inducing cerebral ischemia. A transcutaneous measurement of hemoglobin oxygen saturation (SpO_2) was done using a foot-mounted *pulse oximeter* sensor (Nonin, USA). For continuous mean arterial blood pressure (MABP) measurement, an *Abbocath 24G cannula* (Abott, UK) was inserted into the rat femoral artery and connected to a *Statham pressure transducer* and the *Stoelting measuring set* (Stoelting, USA). During the pMCAO procedure/sham operation, the concentration of hemoglobin and glucose in the blood was determined by glucose and hemoglobin analyzers (HemoCue system, HEMOCUE AB, Sweden). Body temperature was maintained using the *FST thermoregulation system* (Fine Science Tools, USA) consisting of a thermostat, a heating pad, and a rectal temperature probe (**Fig.S1 A**).

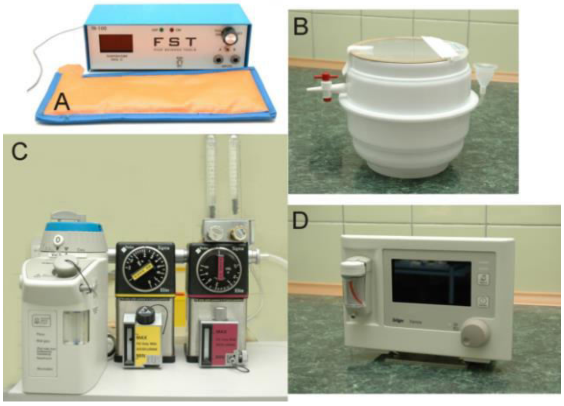

Figure S1. Equipment used for anesthesia and monitoring of the physiological parameters in rats during pMCAO procedure and sham operation. FST thermoregulation system (A), induction chamber into which the rat was placed during induction of anesthesia (B), vaporizer for general anesthesia (C), analyzer for the control of ventilation efficiency and end-tidal sevoflurane concentration (Et_{sevo}) (D).

After the operation, during awakening, respiratory capacity was assessed by continuous measurement of $EtCO_2$ and arterial oxygen saturation (SaO_2). Values below 42 mmHg for $EtCO_2$ and above 95% for SaO_2 along with the proper reactivity were the conditions a condition for transferring the animal to a cage with unlimited access to water and food.

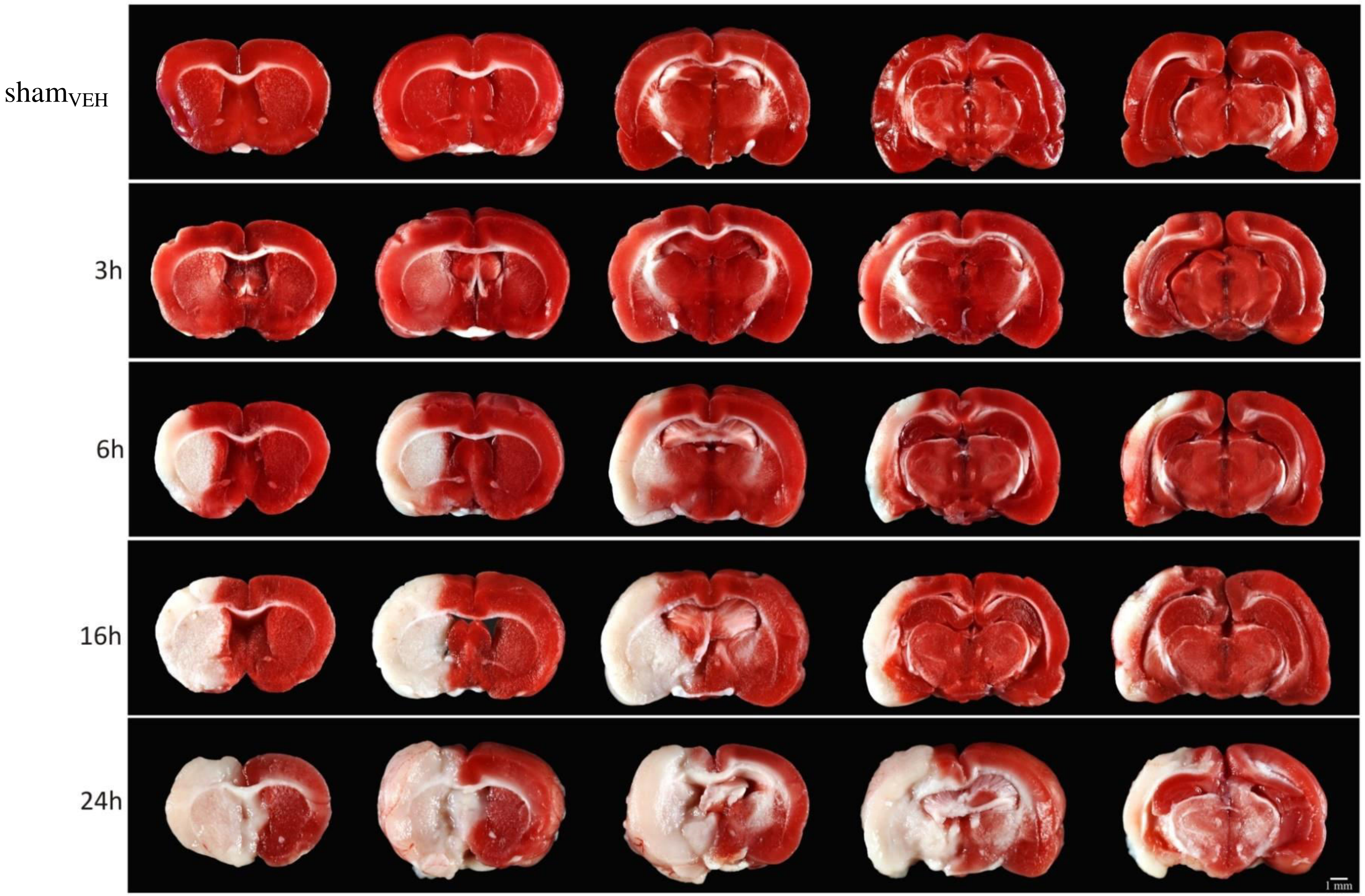

Figure S2. Evolution of the cerebral infarct within the left hemisphere of the rat brain 3, 6, 16, and 24 hours following pMCAO. Coronal sections of the brain stained with 1.5% TTC. sham$_{VEH}$ = rats that underwent operation without pMCAO and were administered with saline solution for 5 days prior to surgery.

Electrophoretic Mobility Shift Assay

Table S1 Sequences of the complementary biotin 3'end-labeled probes used in hybridization reaction

OLIGONUCLEOTIDE SEQUENCES USED IN EMSA	
PROBE 1	5'- GCC TGG GAA AGT CCC CTC AAC T -3'-BIOTIN
PROBE 2	5'- AGT TGA GGG GAC TTT CCC AGG C -3'-BIOTIN

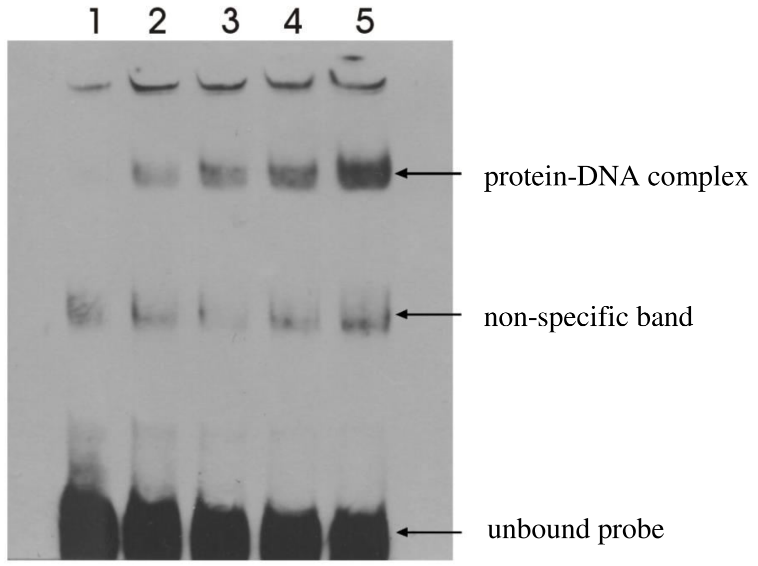

Figure S3. Optimizing a concentration of the nuclear protein extract added to the binding reaction with dsDNA containing the consensus sequence for NF-κB. The protein extract was added to the gel-applied reaction mixture at the final concentration of: 1) 1 µg, 2) 3 µg, 3) 5 µg, 4) 7 µg, and 5) 10 µg. As the protein concentration increases, the activity of NF-κB binding to DNA increases.

Quantitative RT-PCR

qPCR experimental conditions:
initial denaturation: 94°C, 2 minutes

- dsDNA denaturation: 94°C, 45 seconds*
- annealing: 64°C, 45 seconds*
- extension: 72°C, 45 seconds*

* 40 cycles
Each cycle was followed by fluorescence measurement at 75°C.

To exclude SybrGreen-based fluorescence reading of the unspecific qPCR reaction products, we analysed melting curves of amplicons for all samples. The representative melting curves for *Gapdh*, *Bcl-2*, *Bcl-x* i *Actb* are presented in **Fig. S4**.

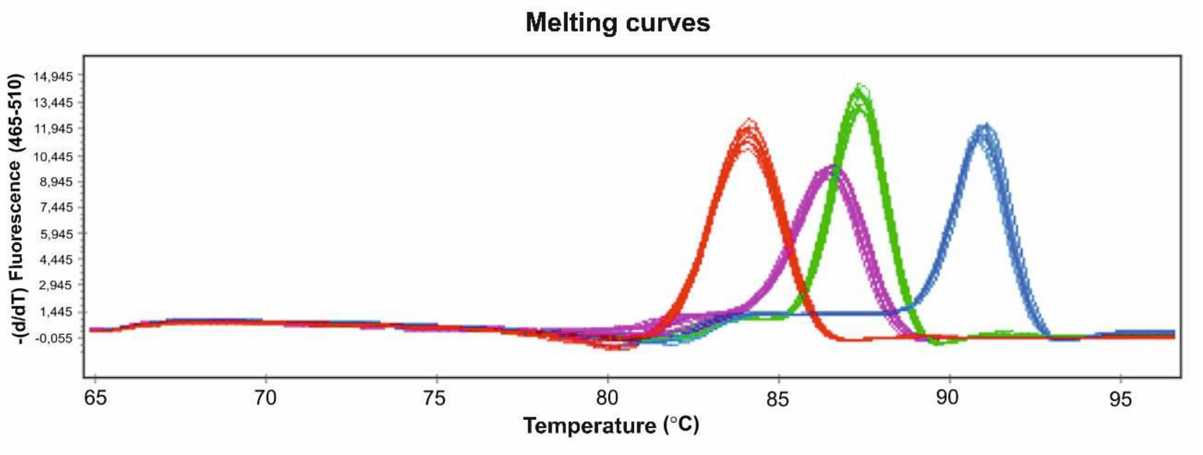

Figure S4. Melting curves of qPCR reaction for *Gapdh* (red), *Bcl-2* (purple), *Bcl-x* (green) i *Actb* (blue) genes. Fluorescence change dynamics -(d/dT) to T enables observation of the quantity and quality of the amplicon and potential presence of unspecific reaction products.

Table S2. Characteristic of the target genes and qPCR primer sequences

GENE/ SYNONYM	PROTEIN	GENBANK NO.	SPECIES	PRIMER SEQUENCE	AMPLICON SIZE (bp)	SOURCE
Puma/ *Bbc3*	BCL2 binding component 3, p53 upregulated modulator of apoptosis (PUMA)	AY157758.1	*Rattus norvegicus*	5'-CCT CAG CCC TCG CTG TCA CCA-3'<br>5'-CCG CCG CTC GTA CTG CGC GTT G-3'	189	&
Noxa/ *Pmaip1*	phorbol-12-myristate-13-acetate-induced protein1, NOXA	NM_001008385.1	*Rattus norvegicus*	5'-GCT CAG CTC AGG AAG ATT GG-3'<br>5'-GGC TTC TTC TCA TCG TGC TC-3'	118	DAiN
Bcl-x/ *Bcl-xl, Bcl2l, bcl-x*	bcl-2-like protein 1, B cell lymphoma like X, bcl2-L-1	NM_001033671.1 isoform 3	*Rattus norvegicus*	5'-AAT GCA GCA GCC GAG AGC CG-3'<br>5'-GGG GAA GGG GCG TGA GTT GC-3'	226	DAiN
Bcl-2/ *Bcl-2, B-cell CLL/lymphoma 2*	B-cell leukemia/lymphoma 2, Bcl2-like protein/ apoptosis regulator BCL-2	NM_016993.1	*Rattus norvegicus*	5'-CTC TCG TCG CTA CCG TCG CG-3'<br>5'-CGT GGC AAA GCG TCC CCT CG-3'	85	DAiN
Actb/*Actx*	β-actin	NM_031144.2	*Rattus norvegicus*	5'-CAA CCT TCT TGC AGC TCC TCC GT-3'<br>5'-TGC CTC TCT TGC TCT GGG CCT C-3'	242	DAiN
Gapdh	glyceraldehyde-3-phosphate dehydrogenase protein (GAPDH)	NM_017008.3	*Rattus norvegicus*	5'-GGG CTC TCT GCT CCT CCC TGT TCT-3'<br>5'-GCC AAA TCC GTT CAC ACC GAC CTT-3'	104	DAiN

& Aleyasin, H. et. al. *J Neurosci* 24, 12 (Mar. 2004), 2963–2973
DAiN - Division of Anatomy and Neurobiology, Medical University of Gdańsk
